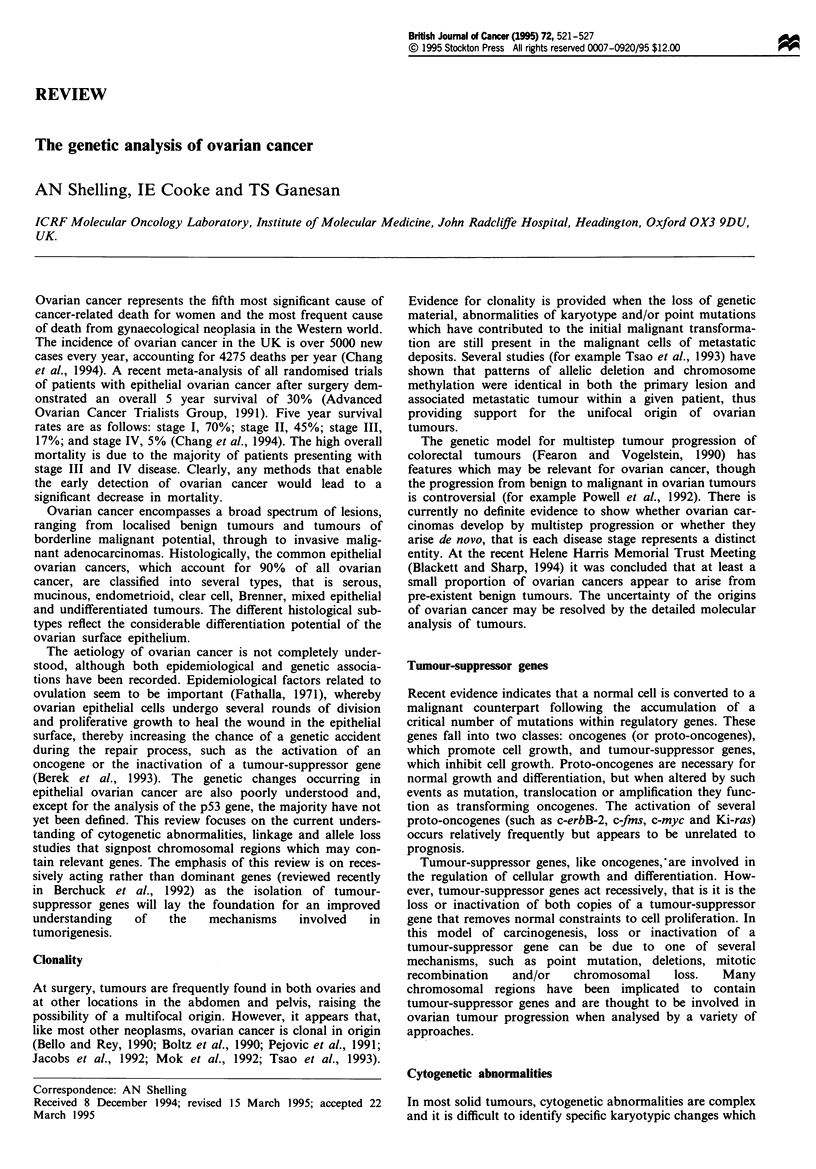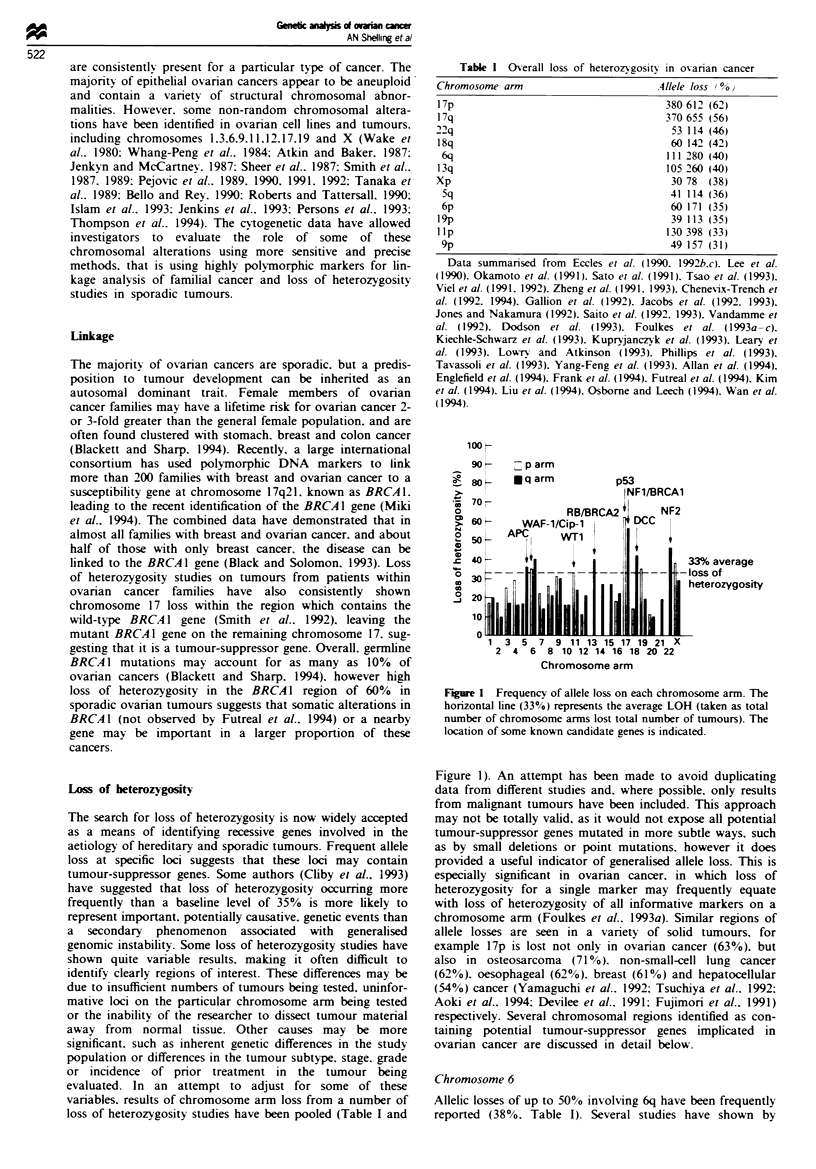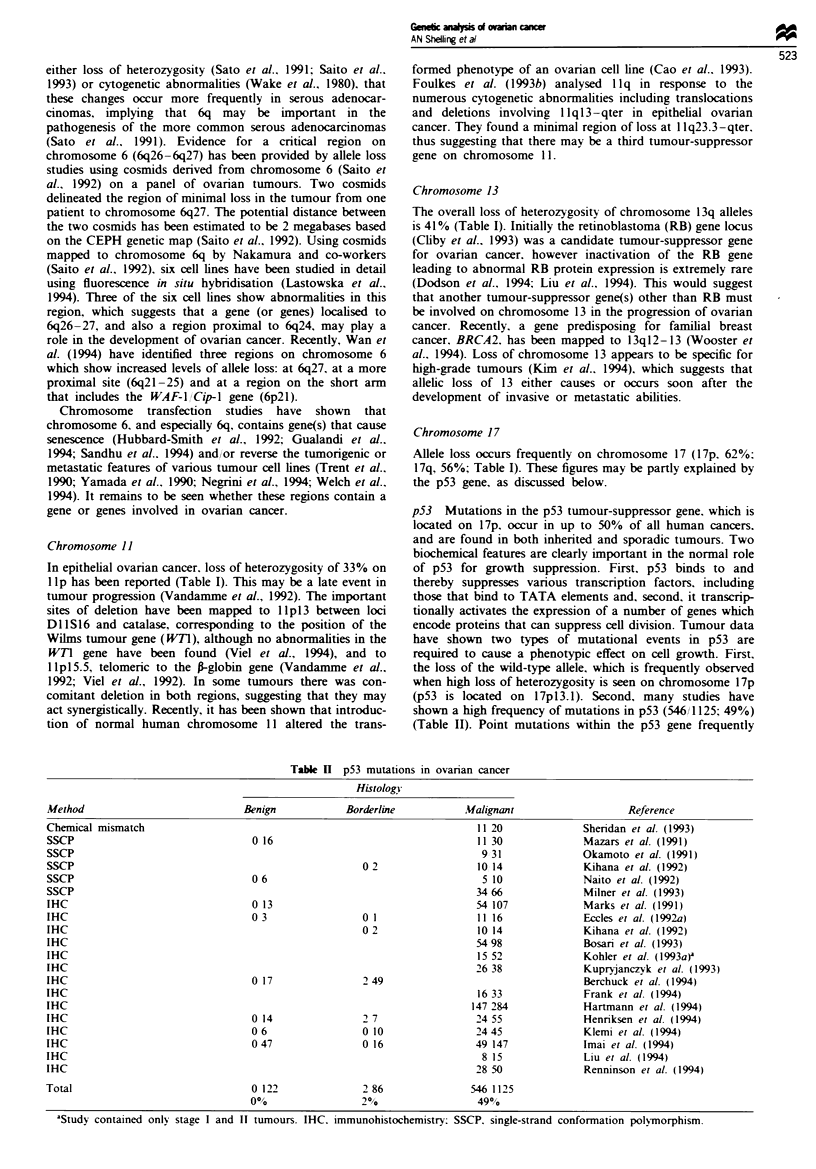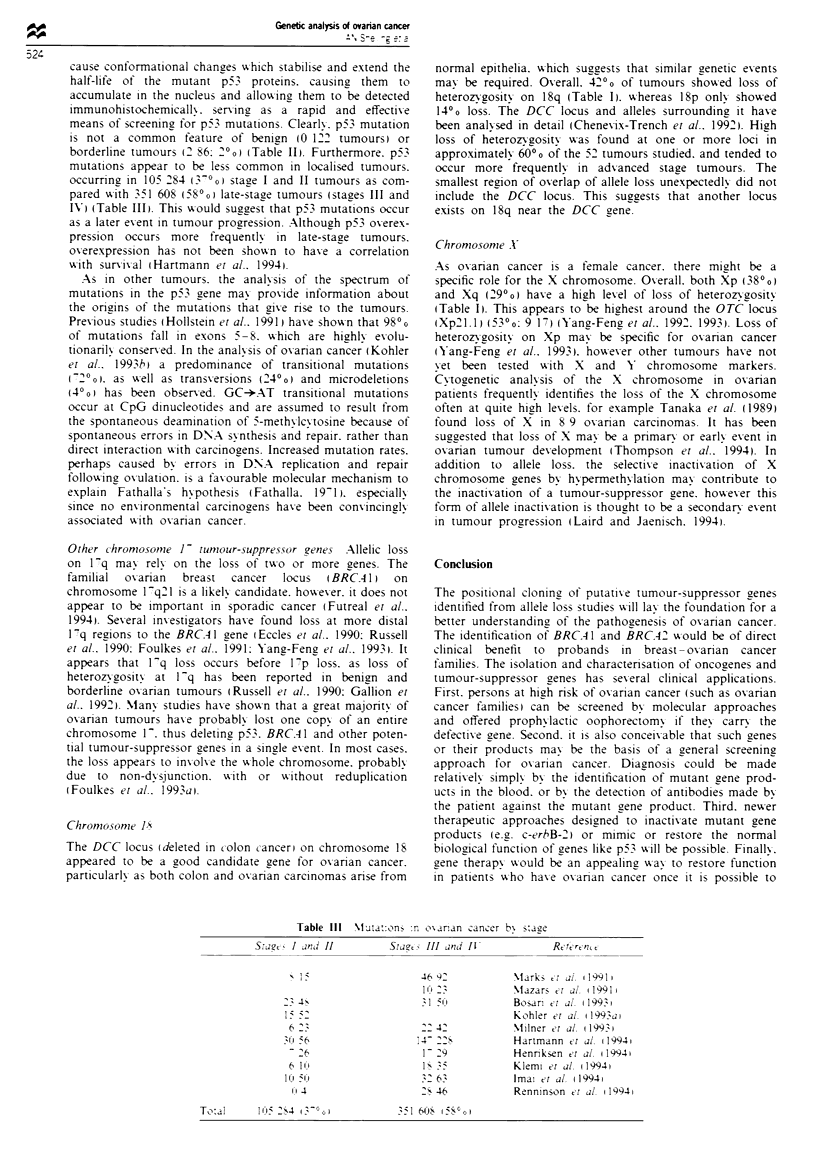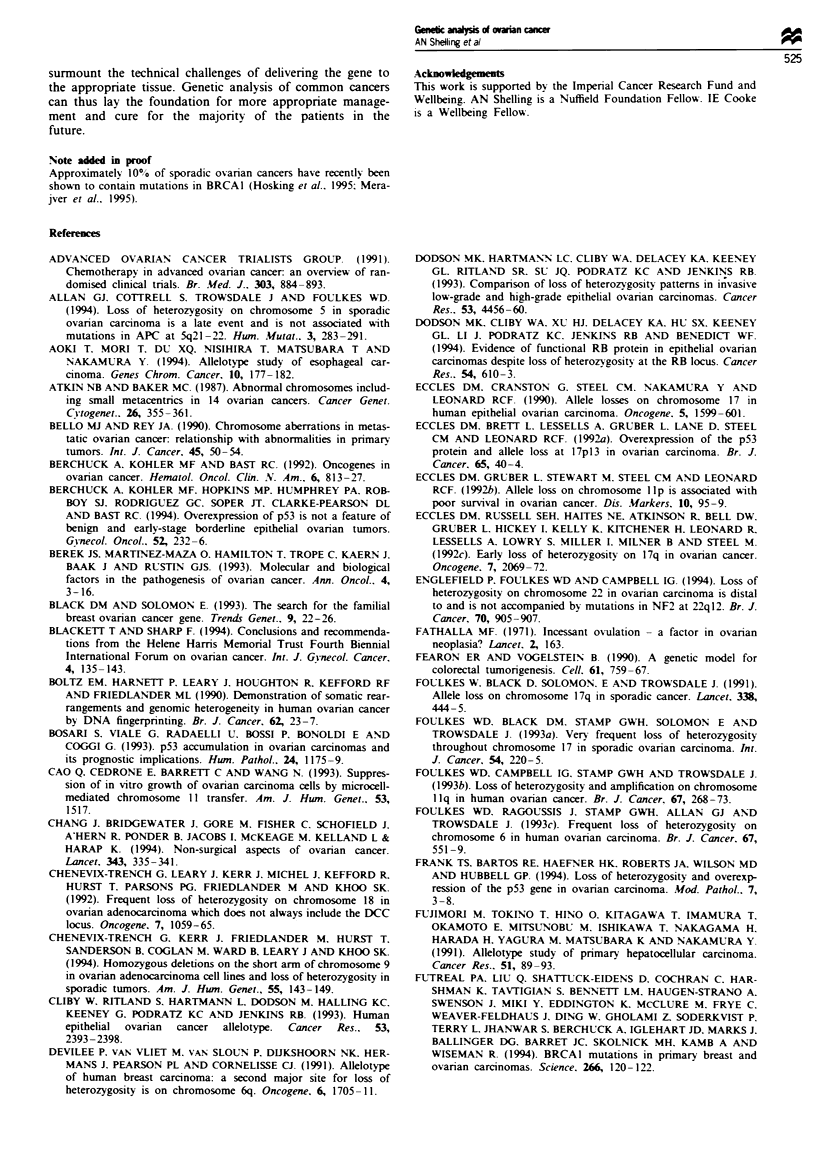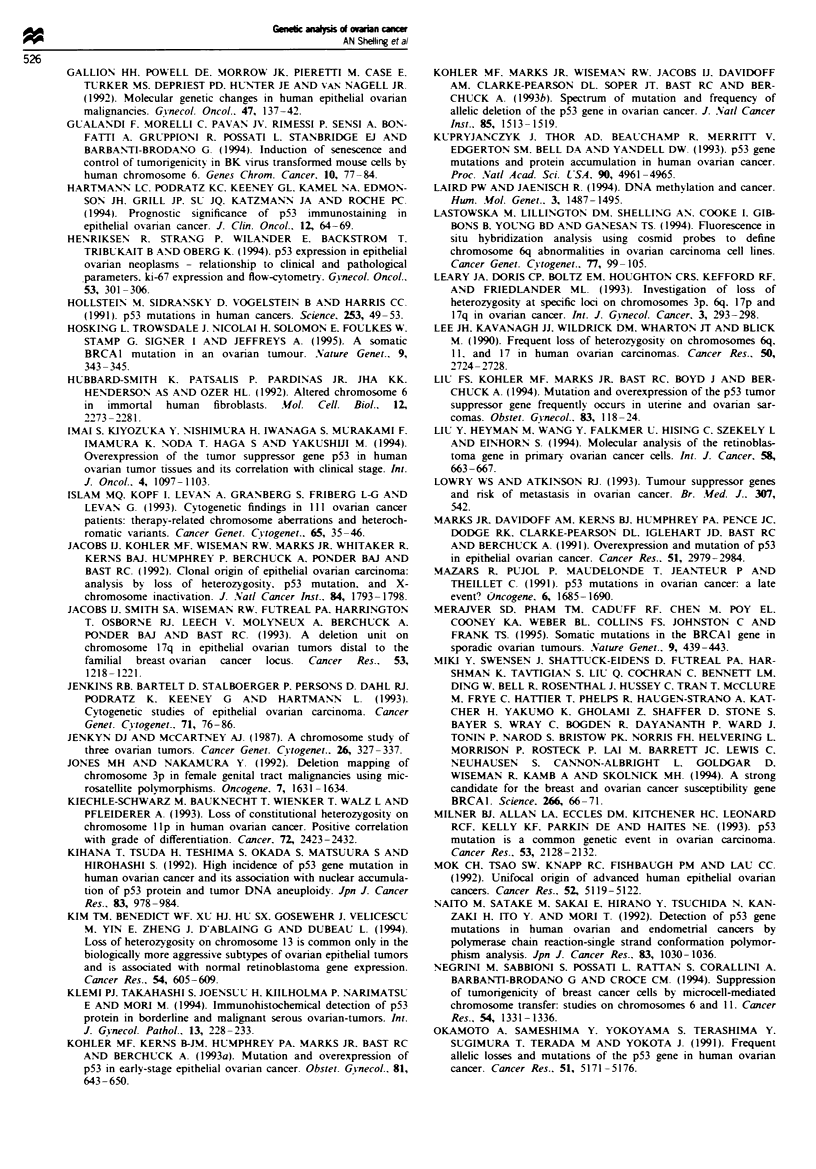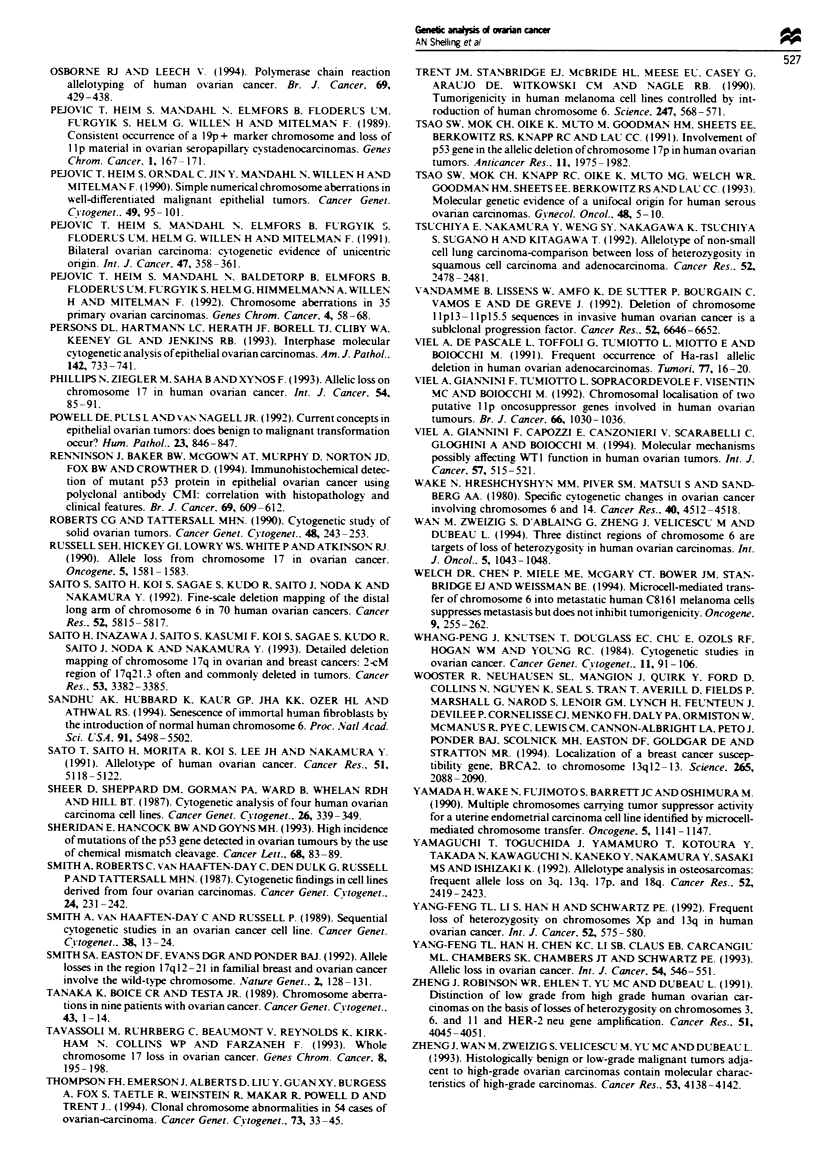# The genetic analysis of ovarian cancer.

**DOI:** 10.1038/bjc.1995.367

**Published:** 1995-09

**Authors:** A. N. Shelling, I. E. Cooke, T. S. Ganesan

**Affiliations:** ICRF Molecular Oncology Laboratory, Institute of Molecular Medicine, John Radcliffe Hospital, Headington, Oxford, UK.


British Journal of Cancer (1995) 72, 521-527
© 1995 Stockton Press All rights reserved 0007-0920/95 $12.00

REVIEW

### The genetic analysis of ovarian cancer

AN Shelling, IE Cooke and TS Ganesan

*ICRF Molecular Oncology Laboratory, Institute of Molecular Medicine, John Radcliffe Hospital, Headington, Oxford OX3 9DU, UK.*

Ovarian cancer represents the fifth most significant cause of cancer-related death for women and the most frequent cause of death from gynaecological neoplasia in the Western world. The incidence of ovarian cancer in the UK is over 5000 new cases every year, accounting for 4275 deaths per year (Chang *et al.*, 1994). A recent meta-analysis of all randomised trials of patients with epithelial ovarian cancer after surgery demonstrated an overall 5 year survival of 30% (Advanced Ovarian Cancer Trialists Group, 1991). Five year survival rates are as follows: stage I, 70%; stage II, 45%; stage III, 17%; and stage IV, 5% (Chang *et al.*, 1994). The high overall mortality is due to the majority of patients presenting with stage III and IV disease. Clearly, any methods that enable the early detection of ovarian cancer would lead to a significant decrease in mortality.

Ovarian cancer encompasses a broad spectrum of lesions, ranging from localised benign tumours and tumours of borderline malignant potential, through to invasive malignant adenocarcinomas. Histologically, the common epithelial ovarian cancers, which account for 90% of all ovarian cancer, are classified into several types, that is serous, mucinous, endometrioid, clear cell, Brenner, mixed epithelial and undifferentiated tumours. The different histological subtypes reflect the considerable differentiation potential of the ovarian surface epithelium.

The aetiology of ovarian cancer is not completely understood, although both epidemiological and genetic associations have been recorded. Epidemiological factors related to ovulation seem to be important (Fathalla, 1971), whereby ovarian epithelial cells undergo several rounds of division and proliferative growth to heal the wound in the epithelial surface, thereby increasing the chance of a genetic accident during the repair process, such as the activation of an oncogene or the inactivation of a tumour-suppressor gene (Berek *et al.*, 1993). The genetic changes occurring in epithelial ovarian cancer are also poorly understood and, except for the analysis of the p53 gene, the majority have not yet been defined. This review focuses on the current understanding of cytogenetic abnormalities, linkage and allele loss studies that signpost chromosomal regions which may contain relevant genes. The emphasis of this review is on recessively acting rather than dominant genes (reviewed recently in Berchuck *et al.*, 1992) as the isolation of tumour-suppressor genes will lay the foundation for an improved understanding of the mechanisms involved in tumorigenesis.

### Clonality

At surgery, tumours are frequently found in both ovaries and at other locations in the abdomen and pelvis, raising the possibility of a multifocal origin. However, it appears that, like most other neoplasms, ovarian cancer is clonal in origin (Bello and Rey, 1990; Boltz *et al.*, 1990; Pejovic *et al.*, 1991; Jacobs *et al.*, 1992; Mok *et al.*, 1992; Tsao *et al.*, 1993). Evidence for clonality is provided when the loss of genetic material, abnormalities of karyotype and/or point mutations which have contributed to the initial malignant transformation are still present in the malignant cells of metastatic deposits. Several studies (for example Tsao *et al.*, 1993) have shown that patterns of allelic deletion and chromosome methylation were identical in both the primary lesion and associated metastatic tumour within a given patient, thus providing support for the unifocal origin of ovarian tumours.

The genetic model for multistep tumour progression of colorectal tumours (Fearon and Vogelstein, 1990) has features which may be relevant for ovarian cancer, though the progression from benign to malignant in ovarian tumours is controversial (for example Powell *et al.*, 1992). There is currently no definite evidence to show whether ovarian carcinomas develop by multistep progression or whether they arise *de novo*, that is each disease stage represents a distinct entity. At the recent Helene Harris Memorial Trust Meeting (Blackett and Sharp, 1994) it was concluded that at least a small proportion of ovarian cancers appear to arise from pre-existent benign tumours. The uncertainty of the origins of ovarian cancer may be resolved by the detailed molecular analysis of tumours.

### Tumour-suppressor genes

Recent evidence indicates that a normal cell is converted to a malignant counterpart following the accumulation of a critical number of mutations within regulatory genes. These genes fall into two classes: oncogenes (or proto-oncogenes), which promote cell growth, and tumour-suppressor genes, which inhibit cell growth. Proto-oncogenes are necessary for normal growth and differentiation, but when altered by such events as mutation, translocation or amplification they function as transforming oncogenes. The activation of several proto-oncogenes (such as c-*erb*B-2, c-*fms*, c-*myc* and Ki-*ras*) occurs relatively frequently but appears to be unrelated to prognosis.

Tumour-suppressor genes, like oncogenes, are involved in the regulation of cellular growth and differentiation. However, tumour-suppressor genes act recessively, that is it is the loss or inactivation of both copies of a tumour-suppressor gene that removes normal constraints to cell proliferation. In this model of carcinogenesis, loss or inactivation of a tumour-suppressor gene can be due to one of several mechanisms, such as point mutation, deletions, mitotic recombination and/or chromosomal loss. Many chromosomal regions have been implicated to contain tumour-suppressor genes and are thought to be involved in ovarian tumour progression when analysed by a variety of approaches.

### Cytogenetic abnormalities

In most solid tumours, cytogenetic abnormalities are complex and it is difficult to identify specific karyotypic changes which

Correspondence: AN Shelling
Received 8 December 1994; revised 15 March 1995; accepted 22 March 1995

are consistently present for a particular type of cancer. The majority of epithelial ovarian cancers appear to be aneuploid and contain a variety of structural chromosomal abnormalities. However, some non-random chromosomal alterations have been identified in ovarian cell lines and tumours, including chromosomes 1,3,6,9,11,12,17,19 and X (Wake *et al.*, 1980; Whang-Peng *et al.*, 1984; Atkin and Baker, 1987; Jenkyn and McCartney, 1987; Sheer *et al.*, 1987; Smith *et al.*, 1987, 1989; Pejovic *et al.*, 1989, 1990, 1991, 1992; Tanaka *et al.*, 1989; Bello and Rey, 1990; Roberts and Tattersall, 1990; Islam *et al.*, 1993; Jenkins *et al.*, 1993; Persons *et al.*, 1993; Thompson *et al.*, 1994). The cytogenetic data have allowed investigators to evaluate the role of some of these chromosomal alterations using more sensitive and precise methods, that is using highly polymorphic markers for linkage analysis of familial cancer and loss of heterozygosity studies in sporadic tumours.

### Linkage

The majority of ovarian cancers are sporadic, but a predisposition to tumour development can be inherited as an autosomal dominant trait. Female members of ovarian cancer families may have a lifetime risk for ovarian cancer 2- or 3-fold greater than the general female population, and are often found clustered with stomach, breast and colon cancer (Blackett and Sharp, 1994). Recently, a large international consortium has used polymorphic DNA markers to link more than 200 families with breast and ovarian cancer to a susceptibility gene at chromosome 17q21, known as *BRCA1*, leading to the recent identification of the *BRCA1* gene (Miki *et al.*, 1994). The combined data have demonstrated that in almost all families with breast and ovarian cancer, and about half of those with only breast cancer, the disease can be linked to the *BRCA1* gene (Black and Solomon, 1993). Loss of heterozygosity studies on tumours from patients within ovarian cancer families have also consistently shown chromosome 17 loss within the region which contains the wild-type *BRCA1* gene (Smith *et al.*, 1992), leaving the mutant *BRCA1* gene on the remaining chromosome 17, suggesting that it is a tumour-suppressor gene. Overall, germline *BRCA1* mutations may account for as many as 10% of ovarian cancers (Blackett and Sharp, 1994), however high loss of heterozygosity in the *BRCA1* region of 60% in sporadic ovarian tumours suggests that somatic alterations in *BRCA1* (not observed by Futreal *et al.*, 1994) or a nearby gene may be important in a larger proportion of these cancers.

### Loss of heterozygosity

The search for loss of heterozygosity is now widely accepted as a means of identifying recessive genes involved in the aetiology of hereditary and sporadic tumours. Frequent allele loss at specific loci suggests that these loci may contain tumour-suppressor genes. Some authors (Cliby *et al.*, 1993) have suggested that loss of heterozygosity occurring more frequently than a baseline level of 35% is more likely to represent important, potentially causative, genetic events than a secondary phenomenon associated with generalised genomic instability. Some loss of heterozygosity studies have shown quite variable results, making it often difficult to identify clearly regions of interest. These differences may be due to insufficient numbers of tumours being tested, uninformative loci on the particular chromosome arm being tested or the inability of the researcher to dissect tumour material away from normal tissue. Other causes may be more significant, such as inherent genetic differences in the study population or differences in the tumour subtype, stage, grade or incidence of prior treatment in the tumour being evaluated. In an attempt to adjust for some of these variables, results of chromosome arm loss from a number of loss of heterozygosity studies have been pooled (Table I and Figure 1). An attempt has been made to avoid duplicating data from different studies and, where possible, only results from malignant tumours have been included. This approach may not be totally valid, as it would not expose all potential tumour-suppressor genes mutated in more subtle ways, such as by small deletions or point mutations, however it does provided a useful indicator of generalised allele loss. This is especially significant in ovarian cancer, in which loss of heterozygosity for a single marker may frequently equate with loss of heterozygosity of all informative markers on a chromosome arm (Foulkes *et al.*, 1993*a*). Similar regions of allele losses are seen in a variety of solid tumours, for example 17p is lost not only in ovarian cancer (63%), but also in osteosarcoma (71%), non-small-cell lung cancer (62%), oesophageal (62%), breast (61%) and hepatocellular (54%) cancer (Yamaguchi *et al.*, 1992; Tsuchiya *et al.*, 1992; Aoki *et al.*, 1994; Devilee *et al.*, 1991; Fujimori *et al.*, 1991) respectively. Several chromosomal regions identified as containing potential tumour-suppressor genes implicated in ovarian cancer are discussed in detail below.

**Table I** Overall loss of heterozygosity in ovarian cancer

| *Chromosome arm* | *Allele loss (%)* |
|---|---|
| 17p | 380 612 (62) |
| 17q | 370 655 (56) |
| 22q | 53 114 (46) |
| 18q | 60 142 (42) |
| 6q | 111 280 (40) |
| 13q | 105 260 (40) |
| Xp | 30 78 (38) |
| 5q | 41 114 (36) |
| 6p | 60 171 (35) |
| 19p | 39 113 (35) |
| 11p | 130 398 (33) |
| 9p | 49 157 (31) |

Data summarised from Eccles *et al.* (1990, 1992*b,c*), Lee *et al.* (1990), Okamoto *et al.* (1991), Sato *et al.* (1991), Tsao *et al.* (1993), Viel *et al.* (1991, 1992), Zheng *et al.* (1991, 1993), Chenevix-Trench *et al.* (1992, 1994), Gallion *et al.* (1992), Jacobs *et al.* (1992, 1993), Jones and Nakamura (1992), Saito *et al.* (1992, 1993), Vandamme *et al.* (1992), Dodson *et al.* (1993), Foulkes *et al.* (1993*a–c*), Kiechle-Schwarz *et al.* (1993), Kupryjanczyk *et al.* (1993), Leary *et al.* (1993), Lowry and Atkinson (1993), Phillips *et al.* (1993), Tavassoli *et al.* (1993), Yang-Feng *et al.* (1993), Allan *et al.* (1994), Englefield *et al.* (1994), Frank *et al.* (1994), Futreal *et al.* (1994), Kim *et al.* (1994), Liu *et al.* (1994), Osborne and Leech (1994), Wan *et al.* (1994).

**Figure 1** Frequency of allele loss on each chromosome arm. The horizontal line (33%) represents the average LOH (taken as total number of chromosome arms lost total number of tumours). The location of some known candidate genes is indicated.

### *Chromosome 6*

Allelic losses of up to 50% involving 6q have been frequently reported (38%, Table I). Several studies have shown by either loss of heterozygosity (Sato *et al*., 1991; Saito *et al*., 1993) or cytogenetic abnormalities (Wake *et al*., 1980), that these changes occur more frequently in serous adenocarcinomas, implying that 6q may be important in the pathogenesis of the more common serous adenocarcinomas (Sato *et al*., 1991). Evidence for a critical region on chromosome 6 (6q26–6q27) has been provided by allele loss studies using cosmids derived from chromosome 6 (Saito *et al*., 1992) on a panel of ovarian tumours. Two cosmids delineated the region of minimal loss in the tumour from one patient to chromosome 6q27. The potential distance between the two cosmids has been estimated to be 2 megabases based on the CEPH genetic map (Saito *et al*., 1992). Using cosmids mapped to chromosome 6q by Nakamura and co-workers (Saito *et al*., 1992), six cell lines have been studied in detail using fluorescence *in situ* hybridisation (Lastowska *et al*., 1994). Three of the six cell lines show abnormalities in this region, which suggests that a gene (or genes) localised to 6q26–27, and also a region proximal to 6q24, may play a role in the development of ovarian cancer. Recently, Wan *et al*. (1994) have identified three regions on chromosome 6 which show increased levels of allele loss: at 6q27, at a more proximal site (6q21–25) and at a region on the short arm that includes the *WAF*-1/*Cip*-1 gene (6p21).

Chromosome transfection studies have shown that chromosome 6, and especially 6q, contains gene(s) that cause senescence (Hubbard-Smith *et al*., 1992; Gualandi *et al*., 1994; Sandhu *et al*., 1994) and/or reverse the tumorigenic or metastatic features of various tumour cell lines (Trent *et al*., 1990; Yamada *et al*., 1990; Negrini *et al*., 1994; Welch *et al*., 1994). It remains to be seen whether these regions contain a gene or genes involved in ovarian cancer.

*Chromosome 11*

In epithelial ovarian cancer, loss of heterozygosity of 33% on 11p has been reported (Table I). This may be a late event in tumour progression (Vandamme *et al*., 1992). The important sites of deletion have been mapped to 11p13 between loci D11S16 and catalase, corresponding to the position of the Wilms tumour gene (*WT1*), although no abnormalities in the *WT1* gene have been found (Viel *et al*., 1994), and to 11p15.5, telomeric to the β-globin gene (Vandamme *et al*., 1992; Viel *et al*., 1992). In some tumours there was concomitant deletion in both regions, suggesting that they may act synergistically. Recently, it has been shown that introduction of normal human chromosome 11 altered the transformed phenotype of an ovarian cell line (Cao *et al*., 1993). Foulkes *et al*. (1993*b*) analysed 11q in response to the numerous cytogenetic abnormalities including translocations and deletions involving 11q13–qter in epithelial ovarian cancer. They found a minimal region of loss at 11q23.3–qter, thus suggesting that there may be a third tumour-suppressor gene on chromosome 11.

*Chromosome 13*

The overall loss of heterozygosity of chromosome 13q alleles is 41% (Table I). Initially the retinoblastoma (RB) gene locus (Cliby *et al*., 1993) was a candidate tumour-suppressor gene for ovarian cancer, however inactivation of the RB gene leading to abnormal RB protein expression is extremely rare (Dodson *et al*., 1994; Liu *et al*., 1994). This would suggest that another tumour-suppressor gene(s) other than RB must be involved on chromosome 13 in the progression of ovarian cancer. Recently, a gene predisposing for familial breast cancer, *BRCA2*, has been mapped to 13q12–13 (Wooster *et al*., 1994). Loss of chromosome 13 appears to be specific for high-grade tumours (Kim *et al*., 1994), which suggests that allelic loss of 13 either causes or occurs soon after the development of invasive or metastatic abilities.

*Chromosome 17*

Allele loss occurs frequently on chromosome 17 (17p, 62%; 17q, 56%; Table I). These figures may be partly explained by the p53 gene, as discussed below.

*p53* Mutations in the p53 tumour-suppressor gene, which is located on 17p, occur in up to 50% of all human cancers, and are found in both inherited and sporadic tumours. Two biochemical features are clearly important in the normal role of p53 for growth suppression. First, p53 binds to and thereby suppresses various transcription factors, including those that bind to TATA elements and, second, it transcriptionally activates the expression of a number of genes which encode proteins that can suppress cell division. Tumour data have shown two types of mutational events in p53 are required to cause a phenotypic effect on cell growth. First, the loss of the wild-type allele, which is frequently observed when high loss of heterozygosity is seen on chromosome 17p (p53 is located on 17p13.1). Second, many studies have shown a high frequency of mutations in p53 (546/1125; 49%) (Table II). Point mutations within the p53 gene frequently

**Table II** p53 mutations in ovarian cancer

| | Histology | | | |
|---|---|---|---|---|
| *Method* | *Benign* | *Borderline* | *Malignant* | *Reference* |
| Chemical mismatch | | | 11/20 | Sheridan *et al*. (1993) |
| SSCP | 0/16 | | 11/30 | Mazars *et al*. (1991) |
| SSCP | | | 9/31 | Okamoto *et al*. (1991) |
| SSCP | | 0/2 | 10/14 | Kihana *et al*. (1992) |
| SSCP | 0/6 | | 5/10 | Naito *et al*. (1992) |
| SSCP | | | 34/66 | Milner *et al*. (1993) |
| IHC | 0/13 | | 54/107 | Marks *et al*. (1991) |
| IHC | 0/3 | 0/1 | 11/16 | Eccles *et al*. (1992*a*) |
| IHC | | 0/2 | 10/14 | Kihana *et al*. (1992) |
| IHC | | | 54/98 | Bosari *et al*. (1993) |
| IHC | | | 15/52 | Kohler *et al*. (1993*a*)[a] |
| IHC | | | 26/38 | Kupryjanczyk *et al*. (1993) |
| IHC | 0/17 | 2/49 | | Berchuck *et al*. (1994) |
| IHC | | | 16/33 | Frank *et al*. (1994) |
| IHC | | | 147/284 | Hartmann *et al*. (1994) |
| IHC | 0/14 | 2/7 | 24/55 | Henriksen *et al*. (1994) |
| IHC | 0/6 | 0/10 | 24/45 | Klemi *et al*. (1994) |
| IHC | 0/47 | 0/16 | 49/147 | Imai *et al*. (1994) |
| IHC | | | 8/15 | Liu *et al*. (1994) |
| IHC | | | 28/50 | Renninson *et al*. (1994) |
| Total | 0/122 | 2/86 | 546/1125 | |
| | 0% | 2% | 49% | |

[a]Study contained only stage I and II tumours. IHC, immunohistochemistry; SSCP, single-strand conformation polymorphism.

cause conformational changes which stabilise and extend the half-life of the mutant p53 proteins, causing them to accumulate in the nucleus and allowing them to be detected immunohistochemically, serving as a rapid and effective means of screening for p53 mutations. Clearly, p53 mutation is not a common feature of benign (0/122 tumours) or borderline tumours (2/86; 2%) (Table II). Furthermore, p53 mutations appear to be less common in localised tumours, occurring in 105/284 (37%) stage I and II tumours as compared with 351/608 (58%) late-stage tumours (stages III and IV) (Table III). This would suggest that p53 mutations occur as a later event in tumour progression. Although p53 overexpression occurs more frequently in late-stage tumours, overexpression has not been shown to have a correlation with survival (Hartmann *et al.*, 1994).

As in other tumours, the analysis of the spectrum of mutations in the p53 gene may provide information about the origins of the mutations that give rise to the tumours. Previous studies (Hollstein *et al.*, 1991) have shown that 98% of mutations fall in exons 5–8, which are highly evolutionarily conserved. In the analysis of ovarian cancer (Kohler *et al.*, 1993*b*) a predominance of transitional mutations (72%), as well as transversions (24%) and microdeletions (4%) has been observed. GC→AT transitional mutations occur at CpG dinucleotides and are assumed to result from the spontaneous deamination of 5-methylcytosine because of spontaneous errors in DNA synthesis and repair, rather than direct interaction with carcinogens. Increased mutation rates, perhaps caused by errors in DNA replication and repair following ovulation, is a favourable molecular mechanism to explain Fathalla's hypothesis (Fathalla, 1971), especially since no environmental carcinogens have been convincingly associated with ovarian cancer.

*Other chromosome 17 tumour-suppressor genes* Allelic loss on 17q may rely on the loss of two or more genes. The familial ovarian breast cancer locus (*BRCA1*) on chromosome 17q21 is a likely candidate, however, it does not appear to be important in sporadic cancer (Futreal *et al.*, 1994). Several investigators have found loss at more distal 17q regions to the *BRCA1* gene (Eccles *et al.*, 1990; Russell *et al.*, 1990; Foulkes *et al.*, 1991; Yang-Feng *et al.*, 1993). It appears that 17q loss occurs before 17p loss, as loss of heterozygosity at 17q has been reported in benign and borderline ovarian tumours (Russell *et al.*, 1990; Gallion *et al.*, 1992). Many studies have shown that a great majority of ovarian tumours have probably lost one copy of an entire chromosome 17, thus deleting p53, *BRCA1* and other potential tumour-suppressor genes in a single event. In most cases, the loss appears to involve the whole chromosome, probably due to non-dysjunction, with or without reduplication (Foulkes *et al.*, 1993*a*).

### *Chromosome 18*

The *DCC* locus (*d*eleted in *c*olon *c*ancer) on chromosome 18 appeared to be a good candidate gene for ovarian cancer, particularly as both colon and ovarian carcinomas arise from normal epithelia, which suggests that similar genetic events may be required. Overall, 42% of tumours showed loss of heterozygosity on 18q (Table I), whereas 18p only showed 14% loss. The *DCC* locus and alleles surrounding it have been analysed in detail (Chenevix-Trench *et al.*, 1992). High loss of heterozygosity was found at one or more loci in approximately 60% of the 52 tumours studied, and tended to occur more frequently in advanced stage tumours. The smallest region of overlap of allele loss unexpectedly did not include the *DCC* locus. This suggests that another locus exists on 18q near the *DCC* gene.

### *Chromosome X*

As ovarian cancer is a female cancer, there might be a specific role for the X chromosome. Overall, both Xp (38%) and Xq (29%) have a high level of loss of heterozygosity (Table I). This appears to be highest around the *OTC* locus (Xp21.1) (53%; 9/17) (Yang-Feng *et al.*, 1992, 1993). Loss of heterozygosity on Xp may be specific for ovarian cancer (Yang-Feng *et al.*, 1993), however other tumours have not yet been tested with X and Y chromosome markers. Cytogenetic analysis of the X chromosome in ovarian patients frequently identifies the loss of the X chromosome often at quite high levels, for example Tanaka *et al.* (1989) found loss of X in 8/9 ovarian carcinomas. It has been suggested that loss of X may be a primary or early event in ovarian tumour development (Thompson *et al.*, 1994). In addition to allele loss, the selective inactivation of X chromosome genes by hypermethylation may contribute to the inactivation of a tumour-suppressor gene, however this form of allele inactivation is thought to be a secondary event in tumour progression (Laird and Jaenisch, 1994).

### Conclusion

The positional cloning of putative tumour-suppressor genes identified from allele loss studies will lay the foundation for a better understanding of the pathogenesis of ovarian cancer. The identification of *BRCA1* and *BRCA2* would be of direct clinical benefit to probands in breast–ovarian cancer families. The isolation and characterisation of oncogenes and tumour-suppressor genes has several clinical applications. First, persons at high risk of ovarian cancer (such as ovarian cancer families) can be screened by molecular approaches and offered prophylactic oophorectomy if they carry the defective gene. Second, it is also conceivable that such genes or their products may be the basis of a general screening approach for ovarian cancer. Diagnosis could be made relatively simply by the identification of mutant gene products in the blood, or by the detection of antibodies made by the patient against the mutant gene product. Third, newer therapeutic approaches designed to inactivate mutant gene products (e.g. c-*erb*B-2) or mimic or restore the normal biological function of genes like p53 will be possible. Finally, gene therapy would be an appealing way to restore function in patients who have ovarian cancer once it is possible to

**Table III** Mutations in ovarian cancer by stage

| | *Stages I and II* | *Stages III and IV* | *Reference* |
|---|---|---|---|
| | 8/15 | 46/92 | Marks *et al.* (1991) |
| | | 10/23 | Mazars *et al.* (1991) |
| | 23/48 | 31/50 | Bosari *et al.* (1993) |
| | 15/52 | | Kohler *et al.* (1993*a*) |
| | 6/23 | 22/42 | Milner *et al.* (1993) |
| | 30/56 | 147/228 | Hartmann *et al.* (1994) |
| | 7/26 | 17/29 | Henriksen *et al.* (1994) |
| | 6/10 | 18/35 | Klemi *et al.* (1994) |
| | 10/50 | 32/63 | Imai *et al.* (1994) |
| | 0/4 | 28/46 | Renninson *et al.* (1994) |
| Total | 105/284 (37%) | 351/608 (58%) | |

surmount the technical challenges of delivering the gene to the appropriate tissue. Genetic analysis of common cancers can thus lay the foundation for more appropriate management and cure for the majority of the patients in the future.

**Note added in proof**

Approximately 10% of sporadic ovarian cancers have recently been shown to contain mutations in BRCA1 (Hosking *et al.*, 1995; Merajver *et al.*, 1995).

**Acknowledgements**

This work is supported by the Imperial Cancer Research Fund and Wellbeing. AN Shelling is a Nuffield Foundation Fellow. IE Cooke is a Wellbeing Fellow.

**References**

ADVANCED OVARIAN CANCER TRIALISTS GROUP. (1991). Chemotherapy in advanced ovarian cancer: an overview of randomised clinical trials. *Br. Med. J.*, **303**, 884-893.

ALLAN GJ. COTTRELL S. TROWSDALE J AND FOULKES WD. (1994). Loss of heterozygosity on chromosome 5 in sporadic ovarian carcinoma is a late event and is not associated with mutations in APC at 5q21-22. *Hum. Mutat.*, **3**, 283-291.

AOKI T. MORI T. DU XQ. NISIHIRA T. MATSUBARA T AND NAKAMURA Y. (1994). Allelotype study of esophageal carcinoma. *Genes Chrom. Cancer*, **10**, 177-182.

ATKIN NB AND BAKER MC. (1987). Abnormal chromosomes including small metacentrics in 14 ovarian cancers. *Cancer Genet. Cytogenet.*, **26**, 355-361.

BELLO MJ AND REY JA. (1990). Chromosome aberrations in metastatic ovarian cancer: relationship with abnormalities in primary tumors. *Int. J. Cancer*, **45**, 50-54.

BERCHUCK A. KOHLER MF AND BAST RC. (1992). Oncogenes in ovarian cancer. *Hematol. Oncol. Clin. N. Am.*, **6**, 813-27.

BERCHUCK A. KOHLER MF. HOPKINS MP. HUMPHREY PA. ROBBOY SJ. RODRIGUEZ GC. SOPER JT. CLARKE-PEARSON DL AND BAST RC. (1994). Overexpression of p53 is not a feature of benign and early-stage borderline epithelial ovarian tumors. *Gynecol. Oncol.*, **52**, 232-6.

BEREK JS. MARTINEZ-MAZA O. HAMILTON T. TROPE C. KAERN J. BAAK J AND RUSTIN GJS. (1993). Molecular and biological factors in the pathogenesis of ovarian cancer. *Ann. Oncol.*, **4**, 3-16.

BLACK DM AND SOLOMON E. (1993). The search for the familial breast ovarian cancer gene. *Trends Genet.*, **9**, 22-26.

BLACKETT T AND SHARP F. (1994). Conclusions and recommendations from the Helene Harris Memorial Trust Fourth Biennial International Forum on ovarian cancer. *Int. J. Gynecol. Cancer*, **4**, 135-143.

BOLTZ EM. HARNETT P. LEARY J. HOUGHTON R. KEFFORD RF AND FRIEDLANDER ML (1990). Demonstration of somatic rearrangements and genomic heterogeneity in human ovarian cancer by DNA fingerprinting. *Br. J. Cancer*, **62**, 23-7.

BOSARI S. VIALE G. RADAELLI U. BOSSI P. BONOLDI E AND COGGI G. (1993). p53 accumulation in ovarian carcinomas and its prognostic implications. *Hum. Pathol.*, **24**, 1175-9.

CAO Q. CEDRONE E. BARRETT C AND WANG N. (1993). Suppression of in vitro growth of ovarian carcinoma cells by microcell-mediated chromosome 11 transfer. *Am. J. Hum. Genet.*, **53**, 1517.

CHANG J. BRIDGEWATER J. GORE M. FISHER C. SCHOFIELD J. A'HERN R. PONDER B. JACOBS I. MCKEAGE M. KELLAND L & HARAP K. (1994). Non-surgical aspects of ovarian cancer. *Lancet*, **343**, 335-341.

CHENEVIX-TRENCH G. LEARY J. KERR J. MICHEL J. KEFFORD R. HURST T. PARSONS PG. FRIEDLANDER M AND KHOO SK. (1992). Frequent loss of heterozygosity on chromosome 18 in ovarian adenocarcinoma which does not always include the DCC locus. *Oncogene*, **7**, 1059-65.

CHENEVIX-TRENCH G. KERR J. FRIEDLANDER M. HURST T. SANDERSON B. COGLAN M. WARD B. LEARY J AND KHOO SK. (1994). Homozygous deletions on the short arm of chromosome 9 in ovarian adenocarcinoma cell lines and loss of heterozygosity in sporadic tumors. *Am. J. Hum. Genet.*, **55**, 143-149.

CLIBY W. RITLAND S. HARTMANN L. DODSON M. HALLING KC. KEENEY G. PODRATZ KC AND JENKINS RB. (1993). Human epithelial ovarian cancer allelotype. *Cancer Res.*, **53**, 2393-2398.

DEVILEE P. VAN VLIET M. VAN SLOUN P. DIJKSHOORN NK. HERMANS J. PEARSON PL AND CORNELISSE CJ. (1991). Allelotype of human breast carcinoma: a second major site for loss of heterozygosity is on chromosome 6q. *Oncogene*, **6**, 1705-11.

DODSON MK. HARTMANN LC. CLIBY WA. DELACEY KA. KEENEY GL. RITLAND SR. SU JQ. PODRATZ KC AND JENKINS RB. (1993). Comparison of loss of heterozygosity patterns in invasive low-grade and high-grade epithelial ovarian carcinomas. *Cancer Res.*, **53**, 4456-60.

DODSON MK. CLIBY WA. XU HJ. DELACEY KA. HU SX. KEENEY GL. LI J. PODRATZ KC. JENKINS RB AND BENEDICT WF. (1994). Evidence of functional RB protein in epithelial ovarian carcinomas despite loss of heterozygosity at the RB locus. *Cancer Res.*, **54**, 610-3.

ECCLES DM. CRANSTON G. STEEL CM. NAKAMURA Y AND LEONARD RCF. (1990). Allele losses on chromosome 17 in human epithelial ovarian carcinoma. *Oncogene*, **5**, 1599-601.

ECCLES DM. BRETT L. LESSELLS A. GRUBER L. LANE D. STEEL CM AND LEONARD RCF. (1992*a*). Overexpression of the p53 protein and allele loss at 17p13 in ovarian carcinoma. *Br. J. Cancer*, **65**, 40-4.

ECCLES DM. GRUBER L. STEWART M. STEEL CM AND LEONARD RCF. (1992*b*). Allele loss on chromosome 11p is associated with poor survival in ovarian cancer. *Dis. Markers*, **10**, 95-9.

ECCLES DM. RUSSELL SEH. HAITES NE. ATKINSON R. BELL DW. GRUBER L. HICKEY I. KELLY K. KITCHENER H. LEONARD R. LESSELLS A. LOWRY S. MILLER I. MILNER B AND STEEL M. (1992*c*). Early loss of heterozygosity on 17q in ovarian cancer. *Oncogene*, **7**, 2069-72.

ENGLEFIELD P. FOULKES WD AND CAMPBELL IG. (1994). Loss of heterozygosity on chromosome 22 in ovarian carcinoma is distal to and is not accompanied by mutations in NF2 at 22q12. *Br. J. Cancer*, **70**, 905-907.

FATHALLA MF. (1971). Incessant ovulation - a factor in ovarian neoplasia? *Lancet*, **2**, 163.

FEARON ER AND VOGELSTEIN B. (1990). A genetic model for colorectal tumorigenesis. *Cell*, **61**, 759-67.

FOULKES W. BLACK D. SOLOMON. E AND TROWSDALE J. (1991). Allele loss on chromosome 17q in sporadic cancer. *Lancet*, **338**, 444-5.

FOULKES WD. BLACK DM. STAMP GWH. SOLOMON E AND TROWSDALE J. (1993*a*). Very frequent loss of heterozygosity throughout chromosome 17 in sporadic ovarian carcinoma. *Int. J. Cancer*, **54**, 220-5.

FOULKES WD. CAMPBELL IG. STAMP GWH AND TROWSDALE J. (1993*b*). Loss of heterozygosity and amplification on chromosome 11q in human ovarian cancer. *Br. J. Cancer*, **67**, 268-73.

FOULKES WD. RAGOUSSIS J. STAMP GWH. ALLAN GJ AND TROWSDALE J. (1993*c*). Frequent loss of heterozygosity on chromosome 6 in human ovarian carcinoma. *Br. J. Cancer*, **67**, 551-9.

FRANK TS. BARTOS RE. HAEFNER HK. ROBERTS JA. WILSON MD AND HUBBELL GP. (1994). Loss of heterozygosity and overexpression of the p53 gene in ovarian carcinoma. *Mod. Pathol.*, **7**, 3-8.

FUJIMORI M. TOKINO T. HINO O. KITAGAWA T. IMAMURA T. OKAMOTO E. MITSUNOBU M. ISHIKAWA T. NAKAGAMA H. HARADA H. YAGURA M. MATSUBARA K AND NAKAMURA Y. (1991). Allelotype study of primary hepatocellular carcinoma. *Cancer Res.*, **51**, 89-93.

FUTREAL PA. LIU Q. SHATTUCK-EIDENS D. COCHRAN C. HARSHMAN K. TAVTIGIAN S. BENNETT LM. HAUGEN-STRANO A. SWENSON J. MIKI Y. EDDINGTON K. MCCLURE M. FRYE C. WEAVER-FELDHAUS J. DING W. GHOLAMI Z. SODERKVIST P. TERRY L. JHANWAR S. BERCHUCK A. IGLEHART JD. MARKS J. BALLINGER DG. BARRET JC. SKOLNICK MH. KAMB A AND WISEMAN R. (1994). BRCA1 mutations in primary breast and ovarian carcinomas. *Science*, **266**, 120-122.

GALLION HH. POWELL DE. MORROW JK. PIERETTI M. CASE E. TURKER MS. DEPRIEST PD. HUNTER JE AND VAN NAGELL JR. (1992). Molecular genetic changes in human epithelial ovarian malignancies. *Gynecol. Oncol.*, **47**, 137–42.

GUALANDI F. MORELLI C. PAVAN JV. RIMESSI P. SENSI A. BONFATTI A. GRUPPIONI R. POSSATI L. STANBRIDGE EJ AND BARBANTI-BRODANO G. (1994). Induction of senescence and control of tumorigenicity in BK virus transformed mouse cells by human chromosome 6. *Genes Chrom. Cancer*, **10**, 77–84.

HARTMANN LC. PODRATZ KC. KEENEY GL. KAMEL NA. EDMONSON JH. GRILL JP. SU JQ. KATZMANN JA AND ROCHE PC. (1994). Prognostic significance of p53 immunostaining in epithelial ovarian cancer. *J. Clin. Oncol.*, **12**, 64–69.

HENRIKSEN R. STRANG P. WILANDER E. BACKSTROM T. TRIBUKAIT B AND OBERG K. (1994). p53 expression in epithelial ovarian neoplasms - relationship to clinical and pathological parameters, ki-67 expression and flow-cytometry. *Gynecol. Oncol.*, **53**, 301–306.

HOLLSTEIN M. SIDRANSKY D. VOGELSTEIN B AND HARRIS CC. (1991). p53 mutations in human cancers. *Science*, **253**, 49–53.

HOSKING L. TROWSDALE J. NICOLAI H. SOLOMON E. FOULKES W. STAMP G. SIGNER I AND JEFFREYS A. (1995). A somatic BRCA1 mutation in an ovarian tumour. *Nature Genet.*, **9**, 343–345.

HUBBARD-SMITH K. PATSALIS P. PARDINAS JR. JHA KK. HENDERSON AS AND OZER HL. (1992). Altered chromosome 6 in immortal human fibroblasts. *Mol. Cell. Biol.*, **12**, 2273–2281.

IMAI S. KIYOZUKA Y. NISHIMURA H. IWANAGA S. MURAKAMI F. IMAMURA K. NODA T. HAGA S AND YAKUSHIJI M. (1994). Overexpression of the tumor suppressor gene p53 in human ovarian tumor tissues and its correlation with clinical stage. *Int. J. Oncol.*, **4**, 1097–1103.

ISLAM MQ. KOPF I. LEVAN A. GRANBERG S. FRIBERG L-G AND LEVAN G. (1993). Cytogenetic findings in 111 ovarian cancer patients: therapy-related chromosome aberrations and heterochromatic variants. *Cancer Genet. Cytogenet.*, **65**, 35–46.

JACOBS IJ. KOHLER MF. WISEMAN RW. MARKS JR. WHITAKER R. KERNS BAJ. HUMPHREY P. BERCHUCK A. PONDER BAJ AND BAST RC. (1992). Clonal origin of epithelial ovarian carcinoma: analysis by loss of heterozygosity, p53 mutation, and X-chromosome inactivation. *J. Natl Cancer Inst.*, **84**, 1793–1798.

JACOBS IJ. SMITH SA. WISEMAN RW. FUTREAL PA. HARRINGTON T. OSBORNE RJ. LEECH V. MOLYNEUX A. BERCHUCK A. PONDER BAJ AND BAST RC. (1993). A deletion unit on chromosome 17q in epithelial ovarian tumors distal to the familial breast ovarian cancer locus. *Cancer Res.*, **53**, 1218–1221.

JENKINS RB. BARTELT D. STALBOERGER P. PERSONS D. DAHL RJ. PODRATZ K. KEENEY G AND HARTMANN L. (1993). Cytogenetic studies of epithelial ovarian carcinoma. *Cancer Genet. Cytogenet.*, **71**, 76–86.

JENKYN DJ AND McCARTNEY AJ. (1987). A chromosome study of three ovarian tumors. *Cancer Genet. Cytogenet.*, **26**, 327–337.

JONES MH AND NAKAMURA Y. (1992). Deletion mapping of chromosome 3p in female genital tract malignancies using microsatellite polymorphisms. *Oncogene*, **7**, 1631–1634.

KIECHLE-SCHWARZ M. BAUKNECHT T. WIENKER T. WALZ L AND PFLEIDERER A. (1993). Loss of constitutional heterozygosity on chromosome 11p in human ovarian cancer. Positive correlation with grade of differentiation. *Cancer*, **72**, 2423–2432.

KIHANA T. TSUDA H. TESHIMA S. OKADA S. MATSUURA S AND HIROHASHI S. (1992). High incidence of p53 gene mutation in human ovarian cancer and its association with nuclear accumulation of p53 protein and tumor DNA aneuploidy. *Jpn J. Cancer Res.*, **83**, 978–984.

KIM TM. BENEDICT WF. XU HJ. HU SX. GOSEWEHR J. VELICESCU M. YIN E. ZHENG J. D'ABLAING G AND DUBEAU L. (1994). Loss of heterozygosity on chromosome 13 is common only in the biologically more aggressive subtypes of ovarian epithelial tumors and is associated with normal retinoblastoma gene expression. *Cancer Res.*, **54**, 605–609.

KLEMI PJ. TAKAHASHI S. JOENSUU H. KIILHOLMA P. NARIMATSU E AND MORI M. (1994). Immunohistochemical detection of p53 protein in borderline and malignant serous ovarian-tumors. *Int. J. Gynecol. Pathol.*, **13**, 228–233.

KOHLER MF. KERNS B-JM. HUMPHREY PA. MARKS JR. BAST RC AND BERCHUCK A. (1993*a*). Mutation and overexpression of p53 in early-stage epithelial ovarian cancer. *Obstet. Gynecol.*, **81**, 643–650.

KOHLER MF. MARKS JR. WISEMAN RW. JACOBS IJ. DAVIDOFF AM. CLARKE-PEARSON DL. SOPER JT. BAST RC AND BERCHUCK A. (1993*b*). Spectrum of mutation and frequency of allelic deletion of the p53 gene in ovarian cancer. *J. Natl Cancer Inst.*, **85**, 1513–1519.

KUPRYJANCZYK J. THOR AD. BEAUCHAMP R. MERRITT V. EDGERTON SM. BELL DA AND YANDELL DW. (1993). p53 gene mutations and protein accumulation in human ovarian cancer. *Proc. Natl Acad. Sci. USA*, **90**, 4961–4965.

LAIRD PW AND JAENISCH R. (1994). DNA methylation and cancer. *Hum. Mol. Genet.*, **3**, 1487–1495.

LASTOWSKA M. LILLINGTON DM. SHELLING AN. COOKE I. GIBBONS B. YOUNG BD AND GANESAN TS. (1994). Fluorescence in situ hybridization analysis using cosmid probes to define chromosome 6q abnormalities in ovarian carcinoma cell lines. *Cancer Genet. Cytogenet.*, **77**, 99–105.

LEARY JA. DORIS CP. BOLTZ EM. HOUGHTON CRS. KEFFORD RF. AND FRIEDLANDER ML. (1993). Investigation of loss of heterozygosity at specific loci on chromosomes 3p, 6q, 17p and 17q in ovarian cancer. *Int. J. Gynecol. Cancer*, **3**, 293–298.

LEE JH. KAVANAGH JJ. WILDRICK DM. WHARTON JT AND BLICK M. (1990). Frequent loss of heterozygosity on chromosomes 6q, 11, and 17 in human ovarian carcinomas. *Cancer Res.*, **50**, 2724–2728.

LIU FS. KOHLER MF. MARKS JR. BAST RC. BOYD J AND BERCHUCK A. (1994). Mutation and overexpression of the p53 tumor suppressor gene frequently occurs in uterine and ovarian sarcomas. *Obstet. Gynecol.*, **83**, 118–24.

LIU Y. HEYMAN M. WANG Y. FALKMER U. HISING C. SZEKELY L AND EINHORN S. (1994). Molecular analysis of the retinoblastoma gene in primary ovarian cancer cells. *Int. J. Cancer*, **58**, 663–667.

LOWRY WS AND ATKINSON RJ. (1993). Tumour suppressor genes and risk of metastasis in ovarian cancer. *Br. Med. J.*, **307**, 542.

MARKS JR. DAVIDOFF AM. KERNS BJ. HUMPHREY PA. PENCE JC. DODGE RK. CLARKE-PEARSON DL. IGLEHART JD. BAST RC AND BERCHUCK A. (1991). Overexpression and mutation of p53 in epithelial ovarian cancer. *Cancer Res.*, **51**, 2979–2984.

MAZARS R. PUJOL P. MAUDELONDE T. JEANTEUR P AND THEILLET C. (1991). p53 mutations in ovarian cancer: a late event? *Oncogene*, **6**, 1685–1690.

MERAJVER SD. PHAM TM. CADUFF RF. CHEN M. POY EL. COONEY KA. WEBER BL. COLLINS FS. JOHNSTON C AND FRANK TS. (1995). Somatic mutations in the BRCA1 gene in sporadic ovarian tumours. *Nature Genet.*, **9**, 439–443.

MIKI Y. SWENSEN J. SHATTUCK-EIDENS D. FUTREAL PA. HARSHMAN K. TAVTIGIAN S. LIU Q. COCHRAN C. BENNETT LM. DING W. BELL R. ROSENTHAL J. HUSSEY C. TRAN T. McCLURE M. FRYE C. HATTIER T. PHELPS R. HAUGEN-STRANO A. KATCHER H. YAKUMO K. GHOLAMI Z. SHAFFER D. STONE S. BAYER S. WRAY C. BOGDEN R. DAYANANTH P. WARD J. TONIN P. NAROD S. BRISTOW PK. NORRIS FH. HELVERING L. MORRISON P. ROSTECK P. LAI M. BARRETT JC. LEWIS C. NEUHAUSEN S. CANNON-ALBRIGHT L. GOLDGAR D. WISEMAN R. KAMB A AND SKOLNICK MH. (1994). A strong candidate for the breast and ovarian cancer susceptibility gene BRCA1. *Science*, **266**, 66–71.

MILNER BJ. ALLAN LA. ECCLES DM. KITCHENER HC. LEONARD RCF. KELLY KF. PARKIN DE AND HAITES NE. (1993). p53 mutation is a common genetic event in ovarian carcinoma. *Cancer Res.*, **53**, 2128–2132.

MOK CH. TSAO SW. KNAPP RC. FISHBAUGH PM AND LAU CC. (1992). Unifocal origin of advanced human epithelial ovarian cancers. *Cancer Res.*, **52**, 5119–5122.

NAITO M. SATAKE M. SAKAI E. HIRANO Y. TSUCHIDA N. KANZAKI H. ITO Y. AND MORI T. (1992). Detection of p53 gene mutations in human ovarian and endometrial cancers by polymerase chain reaction-single strand conformation polymorphism analysis. *Jpn J. Cancer Res.*, **83**, 1030–1036.

NEGRINI M. SABBIONI S. POSSATI L. RATTAN S. CORALLINI A. BARBANTI-BRODANO G AND CROCE CM. (1994). Suppression of tumorigenicity of breast cancer cells by microcell-mediated chromosome transfer: studies on chromosomes 6 and 11. *Cancer Res.*, **54**, 1331–1336.

OKAMOTO A. SAMESHIMA Y. YOKOYAMA S. TERASHIMA Y. SUGIMURA T. TERADA M AND YOKOTA J. (1991). Frequent allelic losses and mutations of the p53 gene in human ovarian cancer. *Cancer Res.*, **51**, 5171–5176.

OSBORNE RJ AND LEECH V. (1994). Polymerase chain reaction allelotyping of human ovarian cancer. *Br. J. Cancer*, **69**, 429-438.

PEJOVIC T. HEIM S. MANDAHL N. ELMFORS B. FLODERUS UM. FURGYIK S. HELM G. WILLEN H AND MITELMAN F. (1989). Consistent occurrence of a 19p+ marker chromosome and loss of 11p material in ovarian seropapillary cystadenocarcinomas. *Genes Chrom. Cancer*, **1**, 167-171.

PEJOVIC T. HEIM S. ORNDAL C. JIN Y. MANDAHL N. WILLEN H AND MITELMAN F. (1990). Simple numerical chromosome aberrations in well-differentiated malignant epithelial tumors. *Cancer Genet. Cytogenet.*, **49**, 95-101.

PEJOVIC T. HEIM S. MANDAHL N. ELMFORS B. FURGYIK S. FLODERUS UM. HELM G. WILLEN H AND MITELMAN F. (1991). Bilateral ovarian carcinoma: cytogenetic evidence of unicentric origin. *Int. J. Cancer*, **47**, 358-361.

PEJOVIC T. HEIM S. MANDAHL N. BALDETORP B. ELMFORS B. FLODERUS UM. FURGYIK S. HELM G. HIMMELMANN A. WILLEN H AND MITELMAN F. (1992). Chromosome aberrations in 35 primary ovarian carcinomas. *Genes Chrom. Cancer*, **4**, 58-68.

PERSONS DL. HARTMANN LC. HERATH JF. BORELL TJ. CLIBY WA. KEENEY GL AND JENKINS RB. (1993). Interphase molecular cytogenetic analysis of epithelial ovarian carcinomas. *Am. J. Pathol.*, **142**, 733-741.

PHILLIPS N. ZIEGLER M. SAHA B AND XYNOS F. (1993). Allelic loss on chromosome 17 in human ovarian cancer. *Int. J. Cancer*, **54**, 85-91.

POWELL DE. PULS L AND VAN NAGELL JR. (1992). Current concepts in epithelial ovarian tumors: does benign to malignant transformation occur? *Hum. Pathol.*, **23**, 846-847.

RENNINSON J. BAKER BW. McGOWN AT. MURPHY D. NORTON JD. FOX BW AND CROWTHER D. (1994). Immunohistochemical detection of mutant p53 protein in epithelial ovarian cancer using polyclonal antibody CMI: correlation with histopathology and clinical features. *Br. J. Cancer*, **69**, 609-612.

ROBERTS CG AND TATTERSALL MHN. (1990). Cytogenetic study of solid ovarian tumors. *Cancer Genet. Cytogenet.*, **48**, 243-253.

RUSSELL SEH. HICKEY GI. LOWRY WS. WHITE P AND ATKINSON RJ. (1990). Allele loss from chromosome 17 in ovarian cancer. *Oncogene*, **5**, 1581-1583.

SAITO S. SAITO H. KOI S. SAGAE S. KUDO R. SAITO J. NODA K AND NAKAMURA Y. (1992). Fine-scale deletion mapping of the distal long arm of chromosome 6 in 70 human ovarian cancers. *Cancer Res.*, **52**, 5815-5817.

SAITO H. INAZAWA J. SAITO S. KASUMI F. KOI S. SAGAE S. KUDO R. SAITO J. NODA K AND NAKAMURA Y. (1993). Detailed deletion mapping of chromosome 17q in ovarian and breast cancers: 2-cM region of 17q21.3 often and commonly deleted in tumors. *Cancer Res.*, **53**, 3382-3385.

SANDHU AK. HUBBARD K. KAUR GP. JHA KK. OZER HL AND ATHWAL RS. (1994). Senescence of immortal human fibroblasts by the introduction of normal human chromosome 6. *Proc. Natl Acad. Sci. USA*, **91**, 5498-5502.

SATO T. SAITO H. MORITA R. KOI S. LEE JH AND NAKAMURA Y. (1991). Allelotype of human ovarian cancer. *Cancer Res.*, **51**, 5118-5122.

SHEER D. SHEPPARD DM. GORMAN PA. WARD B. WHELAN RDH AND HILL BT. (1987). Cytogenetic analysis of four human ovarian carcinoma cell lines. *Cancer Genet. Cytogenet.*, **26**, 339-349.

SHERIDAN E. HANCOCK BW AND GOYNS MH. (1993). High incidence of mutations of the p53 gene detected in ovarian tumours by the use of chemical mismatch cleavage. *Cancer Lett.*, **68**, 83-89.

SMITH A. ROBERTS C. VAN HAAFTEN-DAY C. DEN DULK G. RUSSELL P AND TATTERSALL MHN. (1987). Cytogenetic findings in cell lines derived from four ovarian carcinomas. *Cancer Genet. Cytogenet.*, **24**, 231-242.

SMITH A. VAN HAAFTEN-DAY C AND RUSSELL P. (1989). Sequential cytogenetic studies in an ovarian cancer cell line. *Cancer Genet. Cytogenet.*, **38**, 13-24.

SMITH SA. EASTON DF. EVANS DGR AND PONDER BAJ. (1992). Allele losses in the region 17q12-21 in familial breast and ovarian cancer involve the wild-type chromosome. *Nature Genet.*, **2**, 128-131.

TANAKA K. BOICE CR AND TESTA JR. (1989). Chromosome aberrations in nine patients with ovarian cancer. *Cancer Genet. Cytogenet.*, **43**, 1-14.

TAVASSOLI M. RUHRBERG C. BEAUMONT V. REYNOLDS K. KIRKHAM N. COLLINS WP AND FARZANEH F. (1993). Whole chromosome 17 loss in ovarian cancer. *Genes Chrom. Cancer*, **8**, 195-198.

THOMPSON FH. EMERSON J. ALBERTS D. LIU Y. GUAN XY. BURGESS A. FOX S. TAETLE R. WEINSTEIN R. MAKAR R. POWELL D AND TRENT J.. (1994). Clonal chromosome abnormalities in 54 cases of ovarian-carcinoma. *Cancer Genet. Cytogenet.*, **73**, 33-45.

TRENT JM. STANBRIDGE EJ. McBRIDE HL. MEESE EU. CASEY G. ARAUJO DE. WITKOWSKI CM AND NAGLE RB. (1990). Tumorigenicity in human melanoma cell lines controlled by introduction of human chromosome 6. *Science*, **247**, 568-571.

TSAO SW. MOK CH. OIKE K. MUTO M. GOODMAN HM. SHEETS EE. BERKOWITZ RS. KNAPP RC AND LAU CC. (1991). Involvement of p53 gene in the allelic deletion of chromosome 17p in human ovarian tumors. *Anticancer Res.*, **11**, 1975-1982.

TSAO SW. MOK CH. KNAPP RC. OIKE K. MUTO MG. WELCH WR. GOODMAN HM. SHEETS EE. BERKOWITZ RS AND LAU CC. (1993). Molecular genetic evidence of a unifocal origin for human serous ovarian carcinomas. *Gynecol. Oncol.*, **48**, 5-10.

TSUCHIYA E. NAKAMURA Y. WENG SY. NAKAGAWA K. TSUCHIYA S. SUGANO H AND KITAGAWA T. (1992). Allelotype of non-small cell lung carcinoma-comparison between loss of heterozygosity in squamous cell carcinoma and adenocarcinoma. *Cancer Res.*, **52**, 2478-2481.

VANDAMME B. LISSENS W. AMFO K. DE SUTTER P. BOURGAIN C. VAMOS E AND DE GREVE J. (1992). Deletion of chromosome 11p13-11p15.5 sequences in invasive human ovarian cancer is a subclonal progression factor. *Cancer Res.*, **52**, 6646-6652.

VIEL A. DE PASCALE L. TOFFOLI G. TUMIOTTO L. MIOTTO E AND BOIOCCHI M. (1991). Frequent occurrence of Ha-ras1 allelic deletion in human ovarian adenocarcinomas. *Tumori*, **77**, 16-20.

VIEL A. GIANNINI F. TUMIOTTO L. SOPRACORDEVOLE F. VISENTIN MC AND BOIOCCHI M. (1992). Chromosomal localisation of two putative 11p oncosuppressor genes involved in human ovarian tumours. *Br. J. Cancer*, **66**, 1030-1036.

VIEL A. GIANNINI F. CAPOZZI E. CANZONIERI V. SCARABELLI C. GLOGHINI A AND BOIOCCHI M. (1994). Molecular mechanisms possibly affecting WT1 function in human ovarian tumors. *Int. J. Cancer*, **57**, 515-521.

WAKE N. HRESHCHYSHYN MM. PIVER SM. MATSUI S AND SANDBERG AA. (1980). Specific cytogenetic changes in ovarian cancer involving chromosomes 6 and 14. *Cancer Res.*, **40**, 4512-4518.

WAN M. ZWEIZIG S. D'ABLAING G. ZHENG J. VELICESCU M AND DUBEAU L. (1994). Three distinct regions of chromosome 6 are targets of loss of heterozygosity in human ovarian carcinomas. *Int. J. Oncol.*, **5**, 1043-1048.

WELCH DR. CHEN P. MIELE ME. McGARY CT. BOWER JM. STANBRIDGE EJ AND WEISSMAN BE. (1994). Microcell-mediated transfer of chromosome 6 into metastatic human C8161 melanoma cells suppresses metastasis but does not inhibit tumorigenicity. *Oncogene*, **9**, 255-262.

WHANG-PENG J. KNUTSEN T. DOUGLASS EC. CHU E. OZOLS RF. HOGAN WM AND YOUNG RC. (1984). Cytogenetic studies in ovarian cancer. *Cancer Genet. Cytogenet.*, **11**, 91-106.

WOOSTER R. NEUHAUSEN SL. MANGION J. QUIRK Y. FORD D. COLLINS N. NGUYEN K. SEAL S. TRAN T. AVERILL D. FIELDS P. MARSHALL G. NAROD S. LENOIR GM. LYNCH H. FEUNTEUN J. DEVILEE P. CORNELISSE CJ. MENKO FH. DALY PA. ORMISTON W. McMANUS R. PYE C. LEWIS CM. CANNON-ALBRIGHT LA. PETO J. PONDER BAJ. SCOLNICK MH. EASTON DF. GOLDGAR DE AND STRATTON MR. (1994). Localization of a breast cancer susceptibility gene, BRCA2, to chromosome 13q12-13. *Science*, **265**, 2088-2090.

YAMADA H. WAKE N. FUJIMOTO S. BARRETT JC AND OSHIMURA M. (1990). Multiple chromosomes carrying tumor suppressor activity for a uterine endometrial carcinoma cell line identified by microcell-mediated chromosome transfer. *Oncogene*, **5**, 1141-1147.

YAMAGUCHI T. TOGUCHIDA J. YAMAMURO T. KOTOURA Y. TAKADA N. KAWAGUCHI N. KANEKO Y. NAKAMURA Y. SASAKI MS AND ISHIZAKI K. (1992). Allelotype analysis in osteosarcomas: frequent allele loss on 3q, 13q, 17p, and 18q. *Cancer Res.*, **52**, 2419-2423.

YANG-FENG TL. LI S. HAN H AND SCHWARTZ PE. (1992). Frequent loss of heterozygosity on chromosomes Xp and 13q in human ovarian cancer. *Int. J. Cancer*, **52**, 575-580.

YANG-FENG TL. HAN H. CHEN KC. LI SB. CLAUS EB. CARCANGIU ML. CHAMBERS SK. CHAMBERS JT AND SCHWARTZ PE. (1993). Allelic loss in ovarian cancer. *Int. J. Cancer*, **54**, 546-551.

ZHENG J. ROBINSON WR. EHLEN T. YU MC AND DUBEAU L. (1991). Distinction of low grade from high grade human ovarian carcinomas on the basis of losses of heterozygosity on chromosomes 3, 6, and 11 and HER-2 neu gene amplification. *Cancer Res.*, **51**, 4045-4051.

ZHENG J. WAN M. ZWEIZIG S. VELICESCU M. YU MC AND DUBEAU L. (1993). Histologically benign or low-grade malignant tumors adjacent to high-grade ovarian carcinomas contain molecular characteristics of high-grade carcinomas. *Cancer Res.*, **53**, 4138-4142.